# Neurophysiological oscillatory markers of hypoalgesia in conditioned pain modulation

**DOI:** 10.1097/PR9.0000000000001096

**Published:** 2023-10-23

**Authors:** Hyerang Jin, Bart Witjes, Mathieu Roy, Sylvain Baillet, Cecile C. de Vos

**Affiliations:** a McConnell Brain Imaging Centre, Montreal Neurological Institute, McGill University, Montreal, Canada; b Centre for Pain Medicine, Erasmus University Medical Centre, Rotterdam, the Netherlands; c Department of Psychology, McGill University, Montreal, Canada

**Keywords:** Conditioned pain modulation, Magnetoencephalography, Chronic pain, Pain processing, Pain perception

## Abstract

**Introduction:**

Conditioned pain modulation (CPM) is an experimental procedure that consists of an ongoing noxious stimulus attenuating the pain perception caused by another noxious stimulus. A combination of the CPM paradigm with concurrent electrophysiological recordings can establish whether an association exists between experimentally modified pain perception and modulations of neural oscillations.

**Objectives:**

We aimed to characterize how CPM modifies pain perception and underlying neural oscillations. We also interrogated whether these perceptual and/or neurophysiological effects are distinct in patients affected by chronic pain.

**Methods:**

We presented noxious electrical stimuli to the right ankle before, during, and after CPM induced by an ice pack placed on the left forearm. Seventeen patients with chronic pain and 17 control participants rated the electrical pain in each experimental condition. We used magnetoencephalography to examine the anatomy-specific effects of CPM on the neural oscillatory responses to the electrical pain.

**Results:**

Regardless of the participant groups, CPM induced a reduction in subjective pain ratings and neural responses (beta-band [15–35 Hz] oscillations in the sensorimotor cortex) to electrical pain.

**Conclusion:**

Our findings of pain-induced beta-band activity may be associated with top-down modulations of pain, as reported in other perceptual modalities. Therefore, the reduced beta-band responses during CPM may indicate changes in top-down pain modulations.

## Abstract 2

Supplemental Digital Content is Available in the Text.

We report attenuated subjective pain ratings and neurophysiological responses to electrical pain in both chronic pain and control participants while exposed to conditioned pain modulation.

## 1. Introduction

Neurophysiological responses to nociceptive inputs are inhibited or enhanced by pain modulatory descending pathways, from the cerebrum to the dorsal horns of the spinal cord, in part via the brainstem.^18^ Conditioned pain modulation (CPM) is an experimental procedure that instantiates a “pain inhibits pain” phenomenon, whereby the perception of a noxious test stimulus (TS) is inhibited by the application of another noxious conditioning stimulus (CS) at a heterosegmental site (ie, a region of the body related to a different segment of the spinal cord).^39,40,83^ The diffuse noxious inhibitory control (DNIC) is a hypothesized mechanism of CPM,^39,40^ whereby CS induces ascending nociceptive signals that project to the brainstem, which in turn triggers descending inhibition responses in the spinal cord that attenuate TS pain sensations.^41,47^ According to DNIC, CPM results from bottom-up signals (ie, the CS) triggering a spino-bulbo-spinal loop that induces the perceptual changes of pain. In humans, CPM likely involves both DNIC and top-down modulation (eg, stimulus expectation^52^ and attention^37^) from higher-order brain systems.^7,16,73^ Although the CPM effect is maximal during the application of CS, several studies have found that the effect persists after the removal of CS.^10,27,44^ Therefore, sequential CPM protocols (ie, TS pain hypoalgesia assessed after the termination of CS) can be used to account for potential biases from distraction.^83^

A growing body of research shows clinical relevance of CPM.^44^ Its efficacy is typically reduced in populations experiencing chronic pain, suggesting a disturbance of their pain inhibitory signaling.^15,44,51,69^ However, chronic pain encompasses a considerable variety of phenotypes, and evidence is still missing that CPM has decreased efficacy in patients with chronic low back pain.^50^ There is also a growing interest in using the predictive value of preoperative CPM on the likelihood of developing postoperative chronic pain.^25,66,82^ For all these reasons, advancing the understanding of the neurophysiological mechanisms of CPM is likely to promote its evidence-based adoption for the clinical management of pain.

The modulation of pain-induced neural responses by CPM has been shown with functional magnetic resonance imaging (fMRI) and electrophysiology.^16,23,31,35,49,58^ Conditioned pain modulation reduces pain-induced brain activations in a distributed set of brain regions, including the primary and secondary somatosensory cortices (S1 and S2), anterior cingulate cortex (ACC), insula, and amygdala.^49,58^ Conditioned pain modulation also reduces pain-evoked potentials (EPs) measured at the vertex (Cz) in scalp electrophysiology.^16,23,31,35^ However, if and how event-related spectral perturbations (ERSPs) of ongoing brain oscillations are modulated under CPM remains unknown. Event-related spectral perturbations are typically defined as transient increases (event-related synchronization [ERS]) or decreases (event-related desynchronization [ERD]) of the magnitude of brain electrophysiological activity in a frequency band.^55^ The literature reports that painful stimuli induce (1) a transient suppression of alpha (8–13 Hz) frequency activity (alpha-ERD) across central and posterior brain regions (eg, somatosensory, motor, and visual areas),^32,48,55^ (2) a transient suppression of beta-band (15–30 Hz) activity (beta-ERD) over the sensorimotor cortex,^59,61^ followed by (3) a transient enhancement of beta-band activity (beta-ERS), also over the sensorimotor cortex.^14,24,30^ The functional relevance of these ERSPs remains speculative, with alpha-ERD and beta-ERD possibly reflecting large-scale changes in cortical excitability, and therefore contributing to the alerting function of pain,^32,59^ and beta-ERS possibly signaling top-down components of pain processing.^14^

Here, we used magnetoencephalography (MEG) source imaging^45^ to report the spatiotemporal characteristics of pain-induced ERSPs (alpha-ERD, beta-ERD, and beta-ERS) and describe their changes under CPM, in patients with chronic pain in their lower body and in pain-free control participants.

## 2. Materials and methods

### 2.1. Participants

Seventeen pain-free control participants (HC) and 17 patients (CP) with chronic pain in their low back and/or lower extremity (inclusion criteria) participated. The patients had chronic pain of one or more etiology, with 15 presenting persistent spinal pain syndrome type 2 (aka, failed back surgery syndrome^11^), 3 with diabetic neuropathy, and 1 with neuropathic pain. Exclusion criteria were severe pain in other body parts or any other form of serious decline in general health. HC had no history or current experience of chronic pain. Participants with moderate, nonpainful medical conditions (eg, depression) were not excluded.

Experimental data were collected at the Montreal Neurological Institute (MNI, Montreal, Canada) and the Donders Institute for Brain, Cognition and Behavior (Donders, Nijmegen, the Netherlands). Ethics approval was obtained from the Institutional Review Boards of the MNI and the CMO region Arnhem-Nijmegen. All participants provided informed written consent.

### 2.2. Stimuli

The TS was delivered via transcutaneous electrical stimulations to the right ankle with Ag–AgCl electrodes. One TS trial consisted of five 1-millisecond electrical pulses with 4 milliseconds between 2 consecutive pulses. The stimuli were delivered at randomized intertrial intervals of 6 to 10 seconds to minimize stimulus predictability. Before the experiment, we presented a short series of TS stimuli with ascending and descending intensity to identify the intensity that induced a pain score of 5 on a 0 to 10 scale (0 = no pain, 10 = worst imaginable pain) in each participant.

We delivered the conditioning stimulus with a commercial ice pack (9.5 × 28 cm, containing 500 mL of gel) placed on the participant's left forearm, wrapped in thin fabric to prevent skin damage. The temperature was approximately −10°C, and participants reported moderate pain.

### 2.3. Study protocol

**Figure 1.**
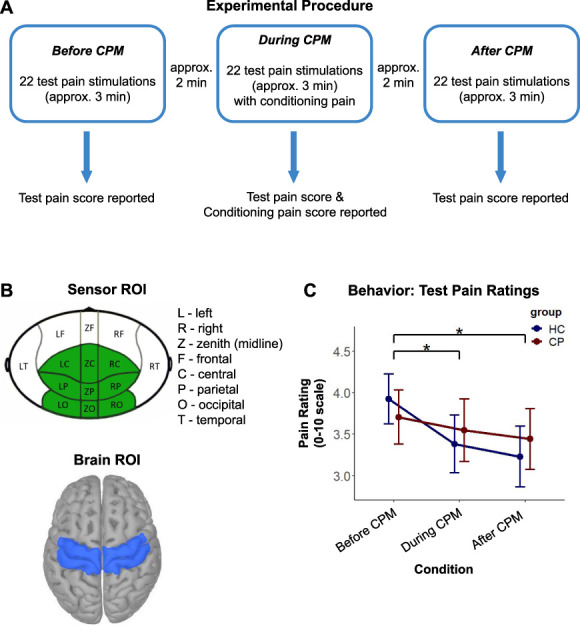
(A) Timeline of the experimental procedure, with one session divided into 3 blocks (*Before*/*During*/*After CPM*). (B) A cluster of MEG sensors defined a region of interest (ROI) over the scalp (in green) for measuring event-related desynchronization in the alpha frequency range (alpha-ERD). This cluster included the central, parietal, and occipital MEG sensors; both recording sites were equipped with identical MEG systems (image modified from 22). The brain ROI used for characterizing event-related desynchronization and synchronization in the beta frequency range (beta-ERD and beta-ERS) is shown in blue. It was anatomically defined across medial and superior lateral sensorimotor cortices, bilaterally. (C) Subjective ratings of test pain stimulation in control and chronic pain participants (mean ± SE). A rank-based ANOVA-type test revealed a main condition effect. A post hoc analysis revealed that the subjective ratings were significantly reduced in the *During CPM* and *After CPM* conditions with respect to the *Before CPM* condition (*P < 0.05). ANOVA, analysis of variance; CPM, conditioned pain modulation; ERS, event-related synchronization; MEG, magnetoencephalography.

The experiment consisted of 3 consecutive blocks (Fig. 1A): *Before CPM* (TS only), *During CPM* (concurrent TS and CS), and *After CPM* (TS only). Each block lasted 3 minutes, with 2-minute breaks in-between. Participants sat still, with eyes open, and focused on a fixation cross presented on a back projection screen. The participants rated average TS intensity on the 0 to 10 scale after each block and rated average CS intensity on the same scale following the *During CPM* block.

### 2.4. Regions of interest

We anticipated alpha-ERD over bilateral central and posterior regions,^59^ and thus, we defined a scalp ROI containing central and posterior MEG sensors (Fig. 1B: sensor ROI). We manually defined a brain ROI to capture beta-ERD and beta-ERS on the cortex of each individual, which included bilateral sensorimotor regions^14,59^ (Fig. 1B: brain ROI).

### 2.5. Data acquisition

Before the experiment, all participants completed questionnaires related to pain evaluation (Brief Pain Inventory [BPI]),^74^ anxiety and depression (Hospital Anxiety and Depression Scale [HADS]),^84^ and maladaptive response to pain (Pain Catastrophizing Scale).^70^

The brain signals were recorded in a passive magnetically shielded room with identical 275-channel CTF MEG systems (Coquitlam, BC, Canada) at each study site. The sampling rate was set to 2400 Hz and third-order gradient compensation was applied for noise reduction. Eye blinks and cardiac activity were recorded from electro-oculograms and electrocardiograms, respectively.

### 2.6. Data analysis

#### 2.6.1. Preprocessing

Magnetoencephalography data were preprocessed and analyzed using Brainstorm.^22^ Poor-quality MEG sensors were excluded from the analysis (0–14 sensors across participants). The TS stimulus artifact (maximum duration 70 ms after stimulus onset) was cropped from the MEG time series and replaced with linear interpolation. The data were then bandpass filtered (1–200 Hz) and notch filtered to remove power line contamination (50, 100, 150, and 200 Hz for Donders, and 60, 120, and 180 Hz for MNI). Signal-space projections^73^ were used to attenuate MEG contamination from eye blinks, cardiac activity, movement (1–7 Hz), and muscle activity (40–240 Hz).

Individual anatomical T1-weighted MRI volumes were co-registered to MEG data when available (in 7 of 34 participants). In other participants, the ICBM152 template was warped to fit the individual's head shape digitized at the time of their MEG visit using a 3D digitizer system (Polhemus Fastrak, Colchester, VT), via affine transformations. The cortical surfaces were tessellated using Freesurfer and reduced to 15,000 vertices. Individual MEG forward models were derived using the overlapping-sphere approach, and source time series were reconstructed using unconstrained minimum-norm estimation.^29^

#### 2.6.2. Spectogram analysis

**Figure 2.**
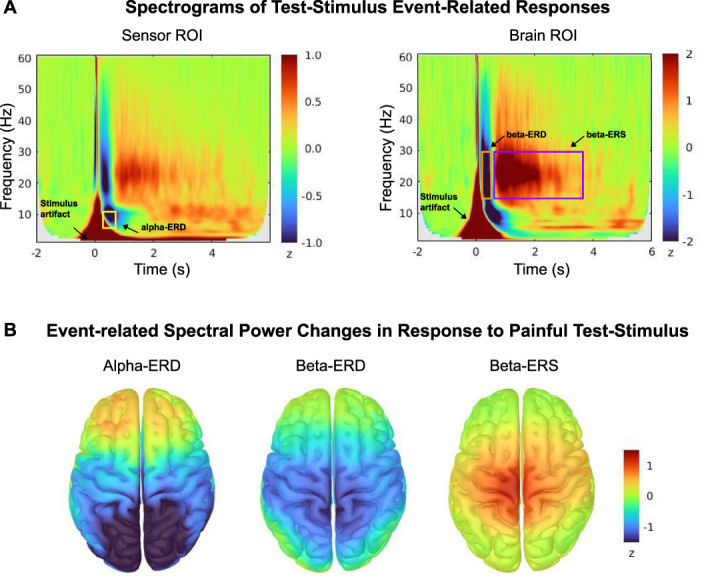
(A) Region of interest (ROI) average time–frequency representations (TFRs). TFRs were averaged across all experimental trials, participants, groups, and conditions across the sensor and brain ROIs shown in Figure 1. Alpha-ERD, beta-ERD, and beta-ERS are highlighted with yellow, orange, and purple boxes, respectively. (B) Whole-brain pain-induced ERSP maps averaged across trials, participants, groups, conditions, and over the time windows identified in (A): alpha-ERD ([0.32, 0.76] seconds), beta-ERD ([0.16, 0.55] seconds), and beta-ERS ([0.61, 3.68] seconds). The frequency range was [8, 13] Hz for the alpha band and [15, 30] Hz for the beta band. The data show pain-induced ERSPs in the hypothesized brain regions: alpha-ERD was expressed over central and posterior brain regions, beta-ERD and beta-ERS localized essentially to sensorimotor cortices. ERD, event-related desynchronization; ERS, event-related synchronization; ERSP, event-related spectral perturbation.

To characterize the ERSPs induced by the TS, we derived time–frequency representations (TFRs) of the signals extracted from the sensor and brain ROIs in every participant. Time–frequency representations in the time range of [−2, 6] seconds (0 second marking TS onset) and the frequency range of [1, 60] Hz were computed with Morlet wavelets (central frequency 1 Hz, time resolution 3 seconds). Time–frequency representation coefficients were then z-scored with respect to prestimulus baseline [−1.5, −0.5] seconds, and averaged across all trials, conditions, and participants, yielding sensor and brain ROI average spectrograms (Fig. 2A).

The time range of the ERSPs in the alpha [8–13 Hz] and beta [15–30 Hz] bands was determined after applying an amplitude threshold of ≥0.5 *z*-score (in absolute value) to the respective ROI average spectrograms (Fig. 2A).

The magnitude of alpha- and beta-band ERSPs was computed by averaging the *z*-scored spectrogram coefficients across the fixed ERSP frequency and time ranges in their respective ROIs. We further measured the duration of the measured ERSPs in all individual participants. To that end, the mean MEG signal magnitude was extracted in each of the predefined frequency ranges and smoothed over time using a moving average *movmean()* function (MATLAB version R2019b) with a window length of 500 milliseconds. We then measured the duration of the ERSP in each individual after applying a threshold of ≥2 *z*-score on the resulting data transforms. Furthermore, the magnitude of an ERSP was extracted at each of the 15,000 brain locations to map the spatial pattern of the ERSP.

We also computed phase-locked EPs in response to the TS. In each participant, MEG time series were averaged across all trials for each study condition and then corrected to the prestimulus baseline interval of [−2, 0] seconds.

#### 2.6.3. Power spectral density analysis

We examined baseline signal power before each TS stimulus delivery. To compare frequency-specific baseline differences between participant groups and experimental conditions, we derived the power spectral density (PSD) of data time series over the prestimulus baseline ([−2, 0] seconds) of each trial (Welch method, one-second sliding time with 50% overlap). Power spectral densities were obtained in the [1, 60] Hz frequency range in each participant's brain ROI and standardized across individuals by dividing the PSD magnitude at each frequency bin by the total signal power across the entire frequency range.

### 2.7. Statistical analysis

Statistical analyses were performed using R^60^ and Brainstorm^71^ with a significance level of 0.05. The 2 study groups were compared for demographics and clinical characteristics using Wilcoxon rank sum tests. We used 2 × 3 nonparametric analysis of variance (ANOVA)-type statistics (ATS) to compare TS pain ratings as well as ERSPs across study groups and conditions, using the *nparLD* package.^53^ The associations between neurophysiological (eg, ERSPs) and behavioral (eg, subjective ratings of the TS) measures were analyzed with Spearman rho correlation coefficients. Cluster-based permutation tests (cluster threshold 0.05, 1000 permutations) were used to compare the TFRs of the ERSPs across study groups and conditions. We also performed nonparametric permutation Student *t* tests (alpha = 0.05, 1000 permutations, false discovery rate [FDR] corrected) for the following analyses: computing the spatial patterns of the ERSP changes induced by CPM and chronic pain, comparing the prestimulus baseline PSDs across the experimental conditions, and comparing the prestimulus baseline characteristics (ie, PSDs, peak frequencies, and total signal power in the alpha and beta frequency bands) between the 2 study groups.

## 3. Results

### 3.1. Patient characteristics

Participant characteristics are summarized in Table 1. The CP and HC groups did not differ in age (*P* = 0.97). However, the CP group featured significantly higher scores in anxiety (*P* < 0.0001, effect size *r* = 0.71), depression (*P* < 0.0001, *r* = 0.76), and pain catastrophizing (*P* < 0.0001, *r* = 0.75). At the time of their MEG visit, 12 patients had pain in the lower back and additional areas (eg, legs, feet, and higher back), 1 patient had lower back pain only, and 4 patients had pain in legs and/or feet.

**Table 1:** Participant characteristics (mean ± SD).

	Patients with chronic pain n = 17	Pain-free control participants n = 17
Participation site	MNI (11), Donders (6)	MNI (11), Donders (6)
Age (y)	50 ± 8	51 ± 10
Sex (male/female)	9/8	10/7
Average pain severity (BPI) (/10)	5.6 ± 2.1*	0 ± 0
Current pain severity (BPI) (/10)	4.8 ± 2.2*	0 ± 0
Chronic pain duration (y)	9.9 ± 9.4*	N/A
Pain locations (participants)	Low back (13) Right (6)/left (12) leg Right (6)/left (8) foot Elsewhere (6)	N/A
Pain etiology (participants)	Persistent Spinal Pain Syndrome Type 2 (PSPS-T2)(13) Diabetic neuropathy (1) PSPS-T2 and diabetic neuropathy (2) Neuropathic pain (1)	N/A
HADS anxiety	8.5 ± 4.3*	1.9 ± 1.2
HADS depression	8.1 ± 3.7*	1.4 ± 2.4
Pain Catastrophizing Scale	23 ± 10*	4.2 ± 4.8
Pain medicine† (taker/nontaker)	11/6	3/14
Nonpain medicine (taker/nontaker)	12/5	5/12

* *P* < 0.0001.

† Opioids, antidepressants, anticonvulsants and nonsteroidal anti-inflammatory drugs (NSAIDs).

BPI, brief pain inventory; HADS, Hospital Anxiety and Depression Scale; MNI, Montreal Neurological Institute.

### 3.2. Behavioral results

Pain intensity and ratings in the 2 study groups are summarized in Table 2. The 2 study groups did not differ in TS intensity (*P* = 0.99) or pain ratings to CS (*P* = 0.45). The experimental condition affected pain ratings of TS (*ATS* [1.95] = 4.76, *P* = 0.009), but there was neither an effect of participant group (*ATS* [1.00] = 0.01, *P* = 0.918) nor an interaction effect (*ATS* [1.95] = 0.70, *P* = 0.491) of TS pain ratings (Fig. 1C). A post hoc analysis revealed that the subjective ratings were significantly reduced in the *During CPM* (*P* adjusted = 0.018) and *After CPM* (*P* adjusted = 0.023) conditions with respect to the *Before CPM* condition.

**Table 2:** Pain intensity and subjective pain ratings (mean ± SD).

	Patients with chronic pain n = 17	Pain-free control participants n = 17
Stimulus intensity (mA, all conditions)	22 ± 15	20 ± 9
Test pain rating: *Before CPM* (/10)	3.7 ± 1.3	3.9 ± 1.2
Test pain rating: *During CPM* (/10)	3.5 ± 1.6	3.4 ± 1.4
Test pain rating: *After CPM* (/10)	3.4 ± 1.5	3.2 ± 1.5
Conditioning pain rating (/10)	3.9 ± 2.2	3.3 ± 2.5

CPM, conditioned pain modulation.

### 3.3. Neurophysiological responses to painful stimuli

We found significant expressions of all 3 hypothesized pain-induced ERSPs (ie, alpha-ERD, beta-ERD, and beta-ERS) across participants and conditions (Fig. 2A) over the following time segments: alpha-ERD ([0.32, 0.76] seconds), beta-ERD ([0.16, 0.55] seconds), and beta-ERS ([0.61, 3.68] seconds). The whole-brain mapping of ERSP effects highlighted the hypothesized brain regions: alpha-ERD was expressed over central and posterior brain regions and beta-ERD and beta-ERS over the sensorimotor cortex, bilaterally (Fig. 2B).

Previous EEG studies have found a reduction in the pain-evoked N1–P1 peak-to-peak amplitude at the vertex (Cz) during CPM.^16,31^ However, such reduction during CPM was not found in our data on visual inspection of the EPs (Supplementary Figure 1, available at http://links.lww.com/PR9/A206). We did not further analyze EPs due to the potential confounding influence of stimulus artifacts (maximum duration of 70 ms poststimulus) on the extraction of early peak amplitudes and latencies.

### 3.4. Effect of conditioned pain modulation and chronic pain on event-related spectral perturbations

**Figure 3.**
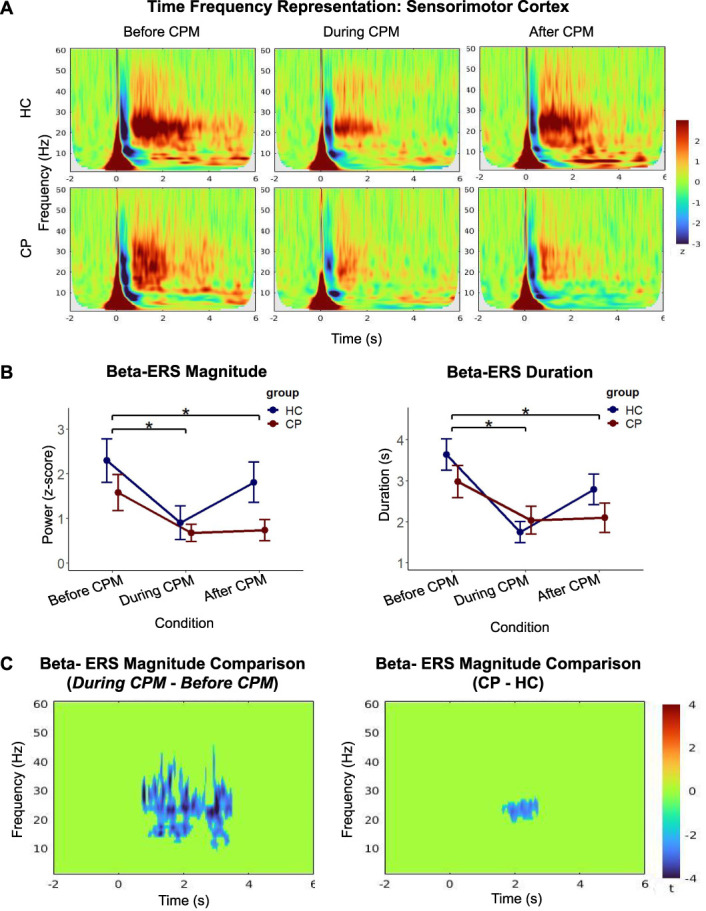
(A) Standardized time–frequency representations (TFRs) of test-stimulus event-related responses of the bilateral sensorimotor cortex before, during, and after CPM in pain-free controls (HC) and in patients with chronic pain (CP). The magnitude and duration of beta-ERS (ie, event-related synchronization in the beta band upon test pain stimulation) were reduced during CPM in both the groups. (B) Sensorimotor beta-ERS magnitude and duration compared across study groups and conditions (mean ± SE). Rank-based ANOVA-type tests revealed main effects of condition in both magnitude and duration of beta-ERS. Post hoc tests showed that beta-ERS magnitude and duration were reduced in the *During CPM* and *After CPM* conditions with respect to the *Before CPM* condition (*P < 0.05) (C) Cluster-corrected TFR of MEG signal magnitude differences in the sensorimotor cortex. The colormap shows the *t*-values of the cluster-based permutation *t* test. The left figure shows the cluster-corrected contrast of TFRs between the *Before CPM* and *During CPM* conditions, for both study groups. Beta-ERS magnitude decreased during CPM (*P* adjusted = 0.006). The right figure shows the cluster-corrected contrasts of TFRs between the HC and CP groups, across all 3 conditions. ANOVA, analysis of variance; CPM, conditioned pain modulation; ERS, event-related synchronization; MEG, magnetoencephalography.

We studied the effect of CPM and chronic pain on ERSPs using ANOVA-type tests and cluster-based permutation tests. We observed a reduction of sensorimotor beta-ERS magnitude and duration during CPM in both groups (Fig. 3A). Rank-based ANOVA-type tests supported these observations (Fig. 3B), showing a significant effect of the study condition on beta-ERS magnitude and duration (ATS [1.98] = 9.06, *P* = 0.0001; ATS [1.99] = 8.46, *P* = 0.0002, respectively). The study group, however, did not have a significant effect on beta-ERS (magnitude: ATS [1.00] = 2.75, *P* = 0.097; duration: ATS [1.00] = 1.32, *P* = 0.251). There was no significant interaction between the effects of condition and group on beta-ERS (magnitude: ATS [1.98] = 0.96, *P* = 0.381; duration: ATS [1.99] = 1.35, *P* = 0.258). Post hoc tests showed that beta-ERS magnitude and duration were reduced in the *During CPM* (*P* adjusted <0.0001; *P* adjusted = 0.0001, respectively) and *After CPM* (*P* adjusted = 0.022; *P* adjusted = 0.028, respectively) conditions with respect to the *Before CPM* condition. Lastly, cluster-based permutation tests further revealed a significant reduction in beta-band magnitude (1–3 seconds poststimulus) during CPM (*P* adjusted = 0.006) (Fig. 3C).

In summary, we found that CPM induced a reduction of beta-ERS in the sensorimotor cortex in both groups. We also found that the study condition or group did not significantly affect alpha-ERD or beta-ERD.

### 3.5. Prestimulus baseline analysis

We then studied whether the observed changes in beta-ERS could be explained by changes in ongoing levels of beta-band activity during the application of CPM as a consequence of CS. Paired nonparametric permutation Student *t* tests did not reveal significant differences between the baseline PSDs of the 3 conditions (Supplementary Figure 2A, available at http://links.lww.com/PR9/A206). Therefore, the reduced beta-ERS during CPM could not be attributed to changes in baseline beta-band activity but rather to changes in beta-band responses to TS.

Moreover, we visually observed a shift in the alpha and beta band peaks towards lower frequencies in patients with chronic pain (Supplementary Figure 2B, available at http://links.lww.com/PR9/A206), consistent with previous resting-state studies in chronic pain populations.^9,78,81^ Despite this observation, independent nonparametric permutation Student *t* tests did not find significant differences in baseline signal power between the HC and CP groups. Additionally, there were no significant differences in peak frequencies nor total signal power in the alpha and beta frequency bands between the 2 groups.

### 3.6. Topography of beta-event–related synchronization

**Figure 4.**
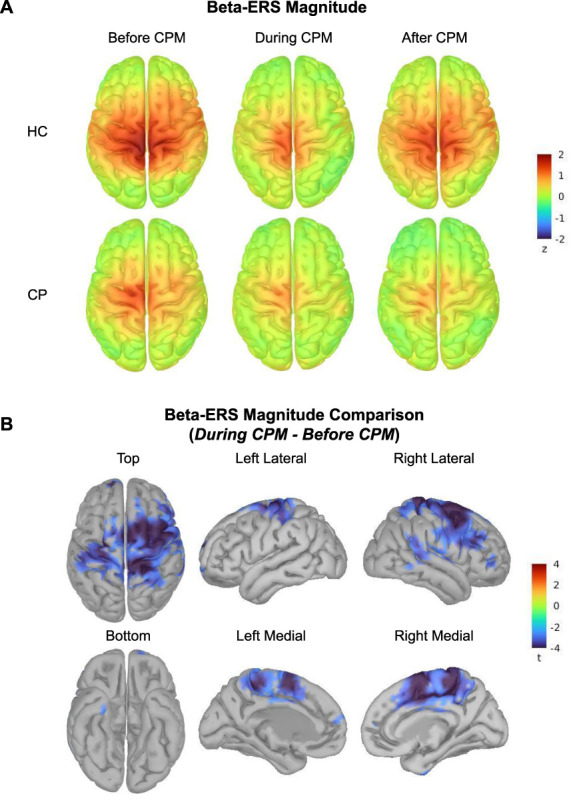
(A) Beta-ERS (time range: [0.61, 3.68] seconds, frequency range: [15, 30] Hz): cortical topography in each group and condition, with respect to prestimulus baseline. In both the groups, beta-ERS (in darker red colors) was attenuated during CPM in and around the sensorimotor cortex. (B) Brain maps of differential beta-ERS magnitudes between the *Before CPM* and *During CPM* conditions, regardless of the study groups. The brain maps depict the *t*-values obtained by paired nonparametric permutation Student *t* tests (alpha = 0.05, 1000 permutations, FDR-corrected for multiple comparisons across 15,000 brain locations). Cortical regions highlighted in blue represent vertices whose beta-ERS magnitude was lower during CPM. CPM, conditioned pain modulation; ERS, event-related synchronization; FDR, false discovery rate.

Beta-ERS was reduced during CPM in the sensorimotor cortex in both study groups (Fig. 4A). The HC group expressed greater sensorimotor beta-ERS magnitude and duration than the CP group in all experimental conditions on average (Fig. 3A). Although we did not find statistically significant group effects in rank-based ANOVA-type tests, we conducted exploratory analyses using permutation Student *t* tests on cortical topography to develop hypotheses for future research. Beta-ERS was attenuated during CPM in the sensorimotor cortex (medial and superior lateral areas) and the supplementary motor area (SMA) (Fig. 4B). Without correcting for multiple comparisons across all 15,000 brain locations, we found a potential group difference between the respective group brain maps of beta-ERS magnitude: HC expressed greater beta-ERS than CP in medial and inferior lateral aspects of the sensorimotor cortex (Supplementary Figure 3, available at http://links.lww.com/PR9/A206). This indication of a group difference remains to be replicated and validated with a larger participant sample size.

### 3.7. Comparison of neural and behavioral effects

The previous analyses demonstrate that CPM induces attenuations in both behavioral (ie, subjective pain ratings) and neural (ie, beta-ERS) measures caused by the TS. However, based on Spearman rho, there was no significant correlation between behavioral and neural measures in response to CPM (*r*_s_ = 0.10, *P* = 0.559, N = 34) (Supplementary Figure 4, available at http://links.lww.com/PR9/A206).

## 4. Discussion

### 4.1. Main findings

Beta-ERS occurred in all 3 experimental conditions (before, during, and after CPM) over the bilateral sensorimotor cortex in both the study groups (ie, CP and HC). We found that both behavioral (subjective pain ratings) and neural (sensorimotor beta-ERS) responses to test pain significantly decreased during CPM, although we did not find a linear association between them. We also found the reduction in beta-ERS to persist after the removal of conditioning pain. There was no main group or interaction effect from the ANOVA-type tests and cluster-based permutation tests, and thus, we cannot draw a conclusion on the difference between HC and CP in their response to CPM.

### 4.2. Functional roles of beta synchronization

The reduction of subjective TS pain ratings induced by CS may result from a combination of DNIC and top-down processes. Such putative neurophysiological supraspinal components of cortical activity related to the CPM effect remain to be characterized.

Beta-ERS has been suggested to be involved in top-down signaling^17,64^ in various tasks such as visual,^6^ auditory,^20^ working memory,^4^ and decision-making^80^ tasks. Therefore, our findings of pain-induced beta-ERS may be associated with top-down cortical modulations in pain processing. Supporting this idea, a study reported that pain-induced beta-ERS in contralateral S1 was increased when participants attended to the pain rather than visual stimuli.^14^ Attention can influence pain perception: attending to a painful stimulus increases the sensation of pain,^77^ and directing attention towards another task or object reduces pain.^76^ Currently, there is mixed evidence of the contribution of attention to the CPM effect. Some studies reported minimal effects of attention,^38,46^ whereas others reported that instructing participants to focus their attention on the CS (vs. the TS) induced a stronger CPM effect.^13,37^ Therefore, one possible interpretation of our findings is that beta-ERS marks attentional modulation involved in pain processing, and their reduction could mark reduced attention toward TS during CPM. Although the participants were not instructed to pay attention to the stimuli, painful stimuli tend to grab attention involuntarily.^42^ Hence, the noxious CS during CPM may have induced an involuntary allocation of attentional resources, potentially leading to lower levels of beta-ERS in response to TS due to competing attentional demands. This interpretation may also explain the partial recovery of beta-ERS after CPM termination, as the removal of the CS may have allowed more attentional resources to be allocated towards the TS.

Furthermore, ERSPs in beta frequencies are commonly observed in motor studies, with movement execution inducing sensorimotor beta-ERD and movement termination inducing sensorimotor beta-ERS.^34,36,56,68^ Consistent with this notion, our finding of pain-induced beta-ERD followed by beta-ERS may reflect motor processing upon nociceptive stimulation. Although the participants were instructed to avoid voluntary movement, the transcutaneous electrical stimulation may have elicited a nociceptive withdrawal reflex (NWR).^2^ This reflex, which results in rapid withdrawal from painful stimuli, may be modulated by top-down input from the sensorimotor cortex.^3,5^ In fact, some studies have found that CPM can reduce NWR amplitude or threshold,^8,72^ complicating the interpretation of CPM-induced cortical activity.

In conclusion, the pain-induced beta-ERS observed in the present study may reflect top-down modulation involved in pain processing, potentially associated with attentional influences. However, we cannot exclude the possibility of beta-ERS reflecting cortical modulation involved in motor processing, particularly related to NWR. Nociceptive withdrawal reflex is believed to primarily involve the thinly myelinated Aδ fibers^1,65^ and is commonly induced by transcutaneous electrical stimulation.^12,21,33,62^ Therefore, future studies using tonic thermal pain as the TS and electromyography to identify any motor involvement could provide a clearer interpretation of pain-induced beta-ERS.

### 4.3. Conditioned pain modulation and chronic pain

Our exploratory analysis showed a trend towards lower beta-ERS in patients with chronic pain than in control participants. Cluster-based comparison of sensorimotor cortex TFRs and whole-brain exploratory analyses revealed that the CP group expressed lower levels of beta-ERS in the medial and inferior lateral sensorimotor cortex. We emphasize that these results are tentative and not supported by the main group effects or interactions reported herein. Our interpretation of potentially lower beta-ERS in chronic pain patients is that they are in a continued CPM state at baseline due to their ongoing experience of chronic pain. Under such circumstances, patients with chronic pain would experience a ceiling effect on CPM whereby experimentally-induced CPM via an external CS does not further reduce the TS-related pain sensation and the associated beta-ERS neurophysiological marker. Therefore, these observations encourage further replication studies to advance our understanding of the potential effect of chronic pain on beta-ERS. Additionally, the selected ROIs for beta-ERS included a broad swath of the medial and superior lateral sensorimotor cortex, which may not be anatomically specific enough and further challenge statistical sensitivity. Another possibility may be that beta-ERS effects occur within a narrower sub-band of the beta frequency range that may also be participant specific.

### 4.4. Alpha and beta desynchronization

We observed global alpha-ERD and broad sensorimotor beta-ERD upon delivering TS, consistent with previous studies. We did not find significant differences between study groups or conditions in terms of these desynchronization effects. Considering that attenuations in alpha and beta oscillations may be related to the relatively higher excitability of neural circuits,^57^ Ploner et al.^59^ proposed that their pain-induced suppression across broad regions may be related to the alerting function of pain and its disruption of ongoing behavior. Relating to the possible interpretation that beta-ERS reflects attentional influences on pain processing, alpha-ERD and beta-ERD may initially alert about the presence of TS, with beta-ERS subsequently signaling the redirection of attentional resources towards that stimulus.

### 4.5. Limitations

There are several limitations with the present data and analyses. Firstly, individual anatomical MRI volumes were not available for most participants, and their individual head shapes were used to warp a standard MRI template. Therefore, we could not account for fully-informed anatomical variability in these participants, which introduced approximations in MEG source mapping. Also, we delivered the TS and CS to the ankle and the forearm, respectively, to maximize the heterotopic effects of CPM.^82^ However, some studies have reported that the application of TS to painful neuropathic sites may enhance^26,63,79^ or reduce CPM efficacy.^43,75^ In the present study, most CP participants experienced chronic pain in their lower extremities, and thus, delivering TS to the painful body sites potentially influenced their CPM response.

Additionally, several patients with chronic pain were on pain medications including opioids (n = 7) and nonsteroidal anti-inflammatory drugs (NSAIDs) (n = 5), which may have affected their endogenous pain inhibitory systems.^54^ A few participants in the HC group also used pain medications (eg, antidepressants and NSAIDs) for nonpainful medical conditions (eg, depression). Participants were not instructed to make changes to their medication use before the experimental session and continued with their usual medication(s). However, there was no group difference in behavioral/neural indicators of CPM between individuals under medication and controls. Additionally, comorbid mental health disorders are common in chronic pain populations,^19^ which may bias group comparison analyses. The inclusion and exclusion criteria in this study aimed to provide a representative sample of the study populations. Although our study groups were not balanced regarding medication use and comorbid medical conditions, we did not solely include HC participants without medical conditions or CP participants without medication use in an attempt to minimize confounding effects. Nevertheless, these confounding factors may still have biased our analyses, which is a limitation of our study.

Also, habituation might play a role in the current study design as it involved 3 consecutive experimental blocks with repeated test stimulus delivery. Whether the reduction in pain ratings and beta-ERS in the *During CPM* block could be an effect of CPM hypoalgesia and/or habituation is unclear. However, we emphasize the partial recovery of beta-ERS in the *After CPM* condition in the HC group, where the experienced CPM effect was substantial. Therefore, the reduction in the magnitude and duration of beta-ERS in the *During CPM* condition cannot be explained by habituation alone.

### 4.6. Conclusion

The present study aimed to characterize pain-induced neurophysiological responses to CPM. Amongst the 3 event-related spectral perturbations that we investigated, only beta-ERS was reduced during CPM. Based on previous findings on the involvement of beta synchronization in top-down brain processes, we discuss the finding of reduced beta-ERS as a manifestation of altered top-down modulation of pain during CPM.

## Disclosures

The authors have no conflict of interest to declare.

## Appendix A. Supplemental digital content

Supplemental digital content associated with this article can be found online at http://links.lww.com/PR9/A206.

## Supplementary Material

SUPPLEMENTARY MATERIAL
