# Supplementary material for: Neurophysiological oscillatory markers of hypoalgesia in conditioned pain modulation
Source: Pain Rep. 2023 Oct 23;8(6):e1096. doi: 10.1097/PR9.0000000000001096 (PMC10597579; doi:10.1097/PR9.0000000000001096)
Supplement: SUPPLEMENTARY MATERIAL [file painreports-8-e1096-s001.pdf]

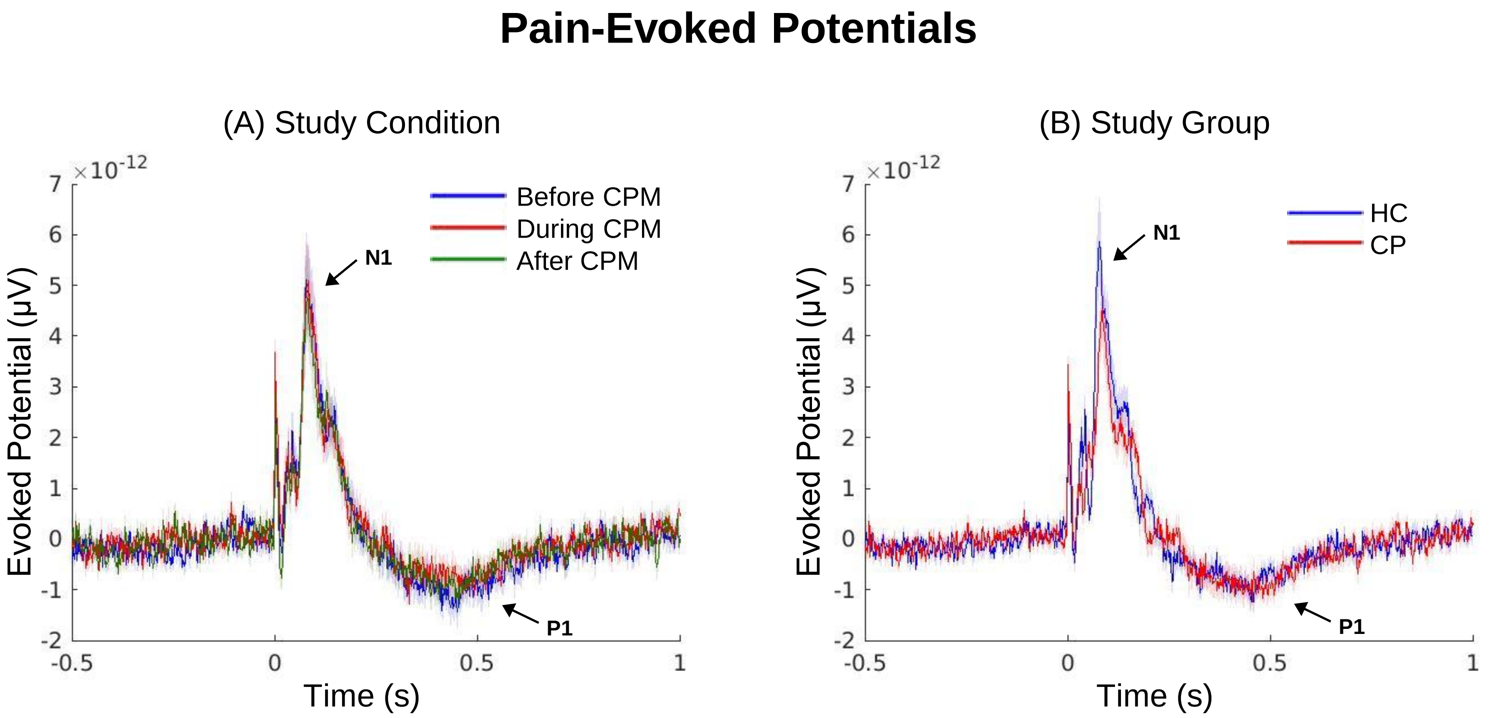

Supplementary Figure 1. Pain-evoked potentials comparing (A) study conditions and (B) study groups in the sensorimotor cortex ROI. The shaded areas represent the standard error. We observed an N1 peak (approx. 80 ms post-stimulus) and a P1 peak (approx. 450 ms post-stimulus). The N1 peak amplitude and latency may have been influenced by stimulus artifact (maximum duration 70 ms post-stimulus).

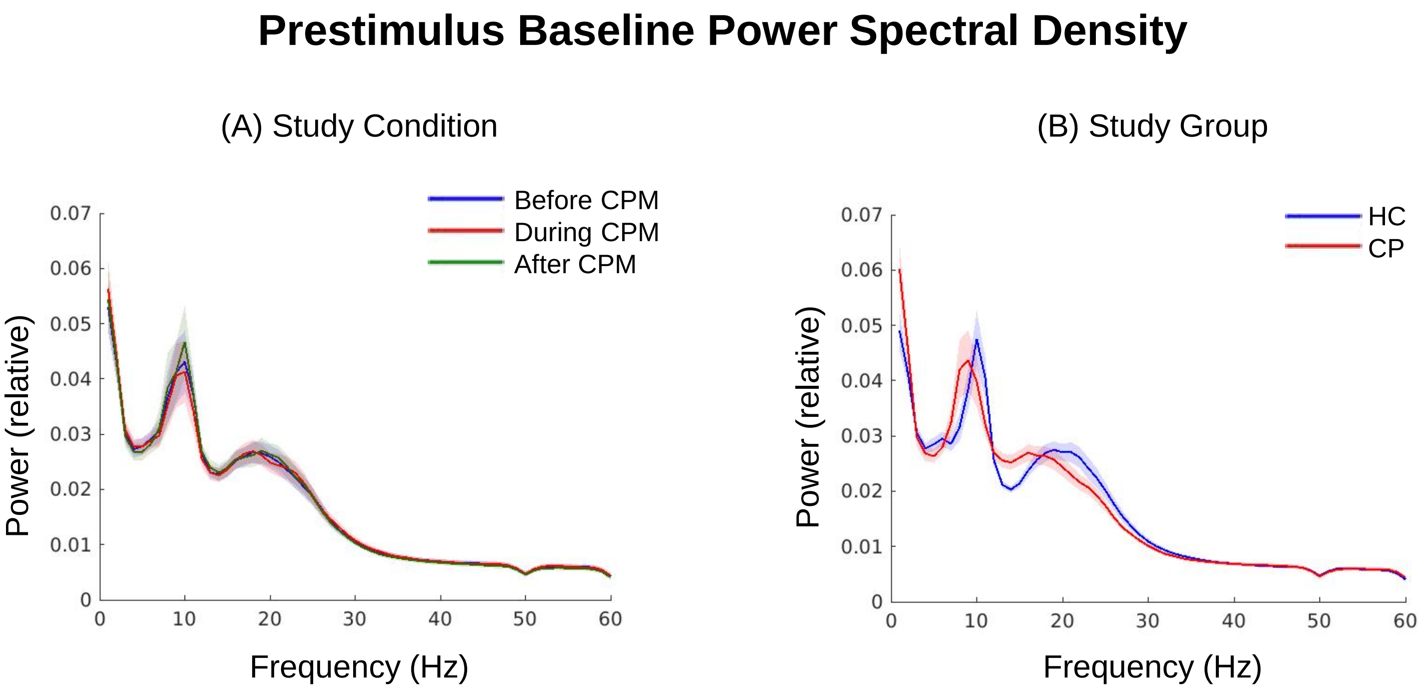

Supplementary Figure 2. Power Spectral Density (PSD) comparing (A) study conditions and (B) study groups during the pre-stimulus baseline [-2,0] s in the sensorimotor cortex ROI. PSDs were obtained using Welch's method (one-second sliding time with 50 % overlap) in the frequency range of [1, 60] Hz in each participant, and the shaded areas represent the standard error. Non-parametric permutation Student's t-tests (alpha = 0.05, 1000 permutations, corrected with false-discovery rate) did not reveal significant differences across the three conditions or between the two groups.

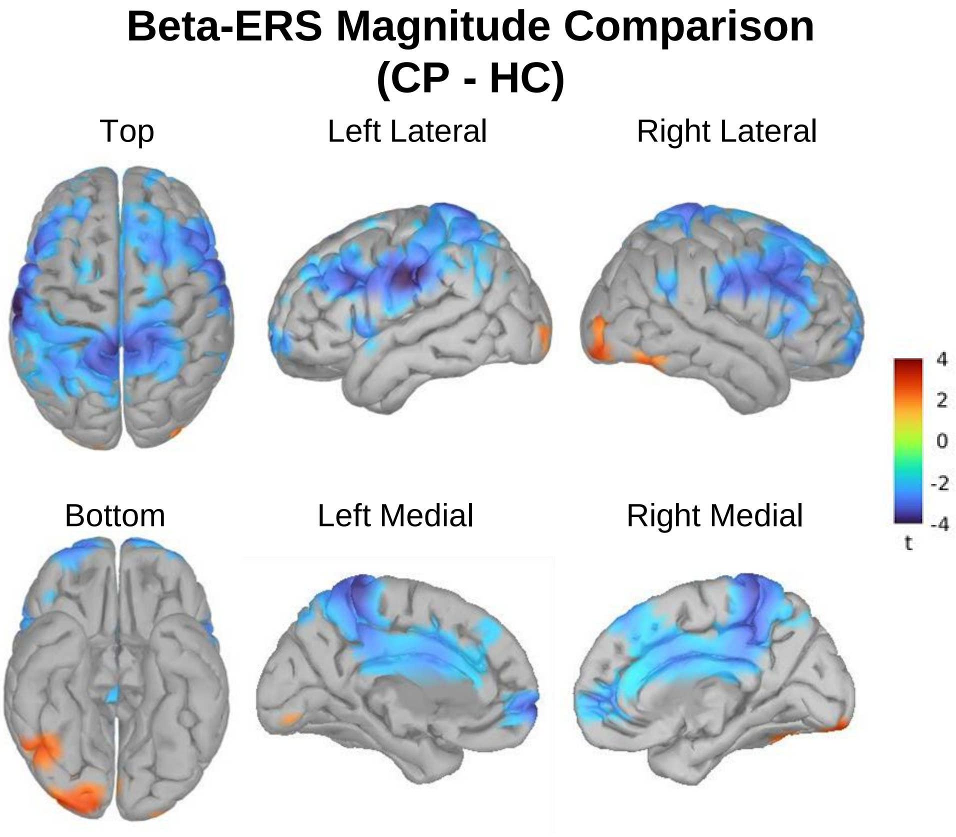

Supplementary Figure 3. Brain topographies comparing beta-ERS magnitude in the two study groups (HC and CP) regardless of the experimental conditions. The brain maps depict t-values obtained from independent permutation Student's t-tests (alpha = 0.05, uncorrected for multiple comparisons across 15000 brain locations, 1000 permutations). Cortical regions highlighted in blue represent brain locations where beta-ERS magnitudes were lower in CP compared to HC.

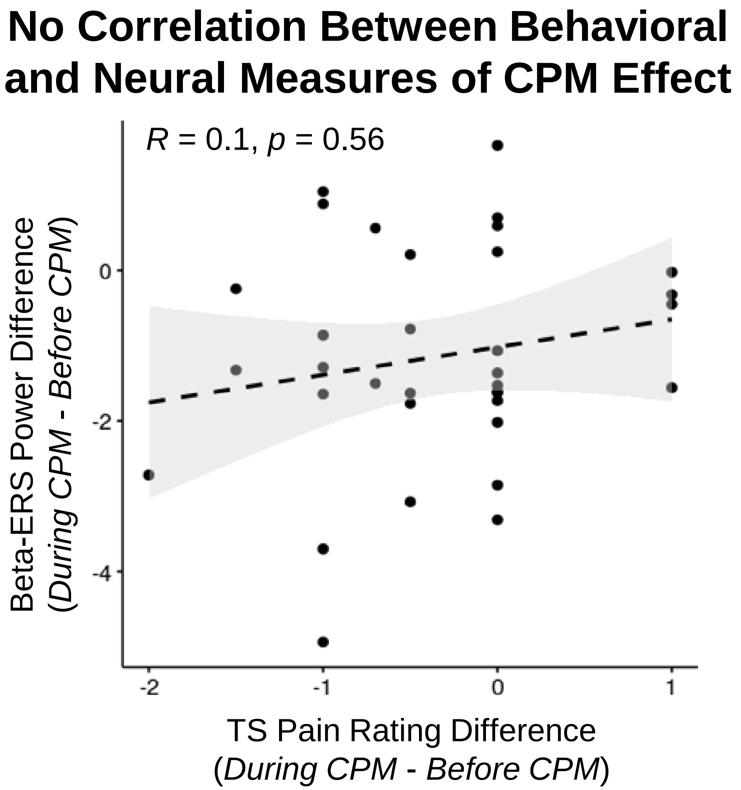

Supplementary Figure 4. No correlation was found between behavioral (i.e., pain ratings to TS) and neural (i.e., beta-ERS) measures of the CPM effect. The regression line is represented in black, and the 95 % confidence interval is shown in grey.
